# Inter-rater and intra-rater reliability of the Chinese version of the short orientation–memory–concentration test in people with stroke

**DOI:** 10.3389/fresc.2025.1614305

**Published:** 2025-08-05

**Authors:** Jiang-Li Zhao, Pei-Ming Chen, Tao Zhang, Hao Xie, Yu-Shu Zhang, Shamay S. M. Ng, Yu-Rong Mao, Dong-Feng Huang

**Affiliations:** ^1^ Department of Rehabilitation Medicine, the First Affiliated Hospital, Sun Yat-sen University, Guangzhou, Guangdong, China; ^2^ Department of Rehabilitation Sciences, The Hong Kong Polytechnic University, Hong Kong SAR, China; ^3^ Department of Rehabilitation Medicine, The Seventh Affiliated Hospital, Sun Yat-sen University, Shenzhen, Guangdong, China

**Keywords:** stroke, rehabilitation, short orientation-memory-concentration test, short blessed test, cognitive impairment, reliability

## Abstract

**Purpose:**

This study aimed to use three statistical methods to investigate the inter-rater and intra-rater reliability of the Chinese version of the Short Orientation-Memory-Concentration Test (C-SOMC) for people who have had a stroke.

**Methods:**

Forty-four participants (31 men and 13 women) with a mean age of 59.05 ± 10.79 years who have had experienced a single episode of stroke were enrolled in this study. To determine the inter-rater reliability of the C-SOMC, the test was administered to each participant on the same day by two raters (A and B) who each had more than 1 year of work experience. To determine intra-rater reliability, rater B re-evaluated 36 of the 44 participants with the C-SOMC on the subsequent day. Intraclass correlation coefficients (ICCs), paired-samples t-tests, and Bland-Altman plots were used to analyze the inter-rater and intra-rater reliability.

**Results:**

The evaluation of inter-rater reliability for the total score and item 1, 4, 5, 6, and 3/7 showed respective ICCs of 0.959, 0.918, 1.000, 0.942, 0.905 and 0.913, indicating excellent inter-rater reliability for the C-SOMC. Item 2 had an ICC of 0.796, indicating moderate to good inter-rater reliability. The evaluation of intra-rater reliability showed an ICC of 0.978 for the total score, and respective ICCs of 1.000, 1.000, 1.000, 0.968, 0.973 and 0.929 for the individual items, indicating excellent intra-rater reliability for the C-SOMC. The paired-samples *t*-test for the C-SOMC showed no statistically significant differences (*P* > 0.05) between ratings made by two different raters or by the same rater on separate occasions. The minimal detectable change value at the 95% confidence threshold (MDC_95_) of the SOMC score was found to be 2.14. Bland-Altman plots showed a mean difference of 0.02 and 95% limits of agreement (95% LOA) ranging from −3.86 to 3.90 for the inter-rater measurements and a mean difference of 0.33 and 95% LOA of −2.71 to 3.37 for the intra-rater measurements.

**Conclusions:**

The C-SOMC demonstrated excellent inter-rater and intra-rater reliability when administered to people who have had a stroke. The C-SOMC may be used to screen for cognitive impairment in people who have had a stroke.

## Introduction

Stroke is the second leading cause of death and the third leading cause of disability worldwide (1). Given the growing global population and advancements in public health and medicine, the number of stroke survivors is increasing annually (2). In 2013, a nationally representative door-to-door survey in China found that the respective age-standardised prevalence and incidence rate of stroke were 1,114.8 per 100,000 people and 246.8 per 100,000 person-years (3). By 2017, stroke had become the leading cause of death, years of life lost, and disability-adjusted life-years in China (4). In addition, in China, the prevalence of stroke continuously rose from 2013 to 2019 (5). Stroke survivors often experience cognitive impairment, physical disability, fatigue, and psychological problems, all of which affect their daily living activities and place huge burdens on their families and society (6). Post-stroke cognitive impairment is the most prevalent clinical syndrome in stroke survivors (7), affecting approximately 20%–80% of this population (2, 8). It can lead to poor recovery of physical functions (9), great restriction in daily activities and social participation, and a lower quality of life (7). Post-stroke cognitive impairment is even associated with the recurrence of ischaemic stroke in high-risk patients (10). However, cognitive impairment is not as noticeable as physical disabilities and can be overlooked by patients and caregivers. Early diagnosis and intervention for cognitive impairment are therefore essential. Assessment for cognitive impairment can be improved by the routine use of validated screening instruments, and very short screening protocols are particularly useful due to their brevity and ease of administration (11).

The Mini-Mental State Examination (MMSE) is the most widely known and used screening tool for evaluating cognition among persons who have had a stroke (12). However, previous studies have reported that use of the MMSE among people with acute and subacute stroke has been limited (13), because of insensitive assessment of executive functioning, working memory, and visual perception. Moreover, many stroke survivors suffer from motor dysfunction of the upper limb, which can affect their ability to perform the necessary drawing task in the MMSE. Furthermore, the MMSE includes a larger number of items and requires a more extended period to administer. Therefore, researchers are searching for alternative screening tools to eliminate the impact of these unrelated factors on the result of cognitive assessment.

The Short Orientation-Memory-Concentration Test (SOMC) is a commonly used screening tool for assessing cognitive function, including the dimensions of orientation, concentration and memory (14, 15). Developed as a modification of the 26-item Information-Memory-Concentration Mental Status Test (16), the SOMC was streamlined to 6 items to enhance clinical utility and ease of administration. The SOMC demonstrates a high degree of correlation (*r* = 0.92) with the full orientation–memory–concentration test and exhibits nearly equivalent sensitivity (15). Additionally, the SOMC test scores are positively correlated with the immediate (Pearson's *r* = 0.68) and delayed (Pearson's *r* = 0.74) paragraph recall scores from the Rivermead Behavioural Memory Test (17), and shows excellent test-retest reliability (*r* = 0.99) in telephone screening of elderly patients (18). Our previous study (19) validated the Chinese version of SOMC for assessing cognitive impairment in stroke patients, demonstrating moderate to good correlations (*r* = 0.57–0.64) with the Chinese version of the MMSE.

The SOMC has been used in various populations such as patients with dementia or Alzheimer's disease, patients given an anesthesia, and healthy older adults (20–26), and previous studies have shown that the SOMC can distinguish between mild, moderate, and severe cognitive deficits (15, 17). The SOMC is brief and can be completed verbally at the patient's bedside without requiring them to write or draw (27), making it particularly friendly for patients with upper limb and hand movement disorders such as stroke survivors. Additionally, the SOMC has excellent test–retest correlation and concurrent validity when used in the telephone screening of elderly patients (18), which allows physicians to efficiently screen the cognitive status of homebound patients. Based on these characteristics, the SOMC is a promising tool for assessing the cognitive status of people who have had a stroke. However, to the best of our knowledge, no further studies investigated the other psychometric properties of SOMC in people with stroke.

Concise and efficient screening instruments such as the Short Orientation-Memory-Concentration (SOMC) test are essential for assessing cognitive function in large populations. The SOMC has already been translated into Dutch (28) and German (29). However, such instruments are currently lacking in China. To allow the use of the SOMC for assessing the cognitive function of Chinese individuals, there is a significant clinical need for a translated Chinese version of the SOMC (C-SOMC) and an investigation of the psychometric properties of this C-SOMC in Chinese population. We therefore translated the original SOMC into Chinese using a translation–back translation procedure and explored its concurrent validity in our previous study (19). Despite this, other important psychometric properties remain unexplored. To facilitate the use of the SOMC for assessing cognitive function in Chinese individuals, there is a significant clinical need for a comprehensive investigation of its psychometric properties in the Chinese population. In addition, to the best of our knowledge, the reliability of the SOMC has not yet been evaluated for use among people who have had a stroke. In this study, the inter-rater and intra-rater reliability of the C-SOMC during stroke population were explored using intra-class correlation coefficient (ICC), paired sample test and Bland-Altman plots.

## Materials and methods

### Study design

This study used a cross-sectional design. The participants' demographic information and major comorbidity data were collected from medical records. This study was approved by the Human Subjects Ethics Subcommittee of the First Affiliated Hospital of Sun Yat-sen University in China (Ethics Number: [2014]88). Informed written consent was obtained from all the participants.

### Participants

The inclusion criteria for the participants were as follows: (1) had a first stroke with unilateral hemiparetic lesions confirmed by magnetic resonance imaging or computed tomography; (2) an interval of ≥5 days after the stroke; (3) aged of 18–80 years; (4) Glasgow coma scale score of 15; (5) no severe deficits in communication; (6) did not have delirium; and (7) able to give informed consent. Patients were excluded if they: (1) were unable to complete assessments due to medical instability; (2) were diagnosed with other neurological diseases that can affect cognitive function; and (3) had taken medication for mental illness.

### Outcome measure

#### The Chinese version of the short orientation–memory–concentration test (C-SOMC)

The C-SOMC consists of 6 items and is used to assess cognitive function along the domains of orientation, concentration and memory. The C-SOMC test is presented in Appendices 1 and 2. Item 6 of the SOMC originally requires reciting the 12 months in reverse order. In Chinese culture, however, reciting months backwards is equivalent to counting backwards from 12 to 1, a task already covered by item 5 (counting backwards from 20 to 1). In C-SOMC, we therefore replaced the months with the twelve Chinese zodiac animals—rat, ox, tiger, rabbit, dragon, snake, horse, goat, monkey, rooster, dog and pig—which are universally familiar in China. Like the SOMC, the total score for the C-SOMC ranges between 0 and 28, with higher scores indicating better cognitive function (30). The scores are categorized as follows: 24–28 = normal cognition; 19–23 = questionable impairment, and ≤18 = dementia (31). The breakdown of scores for each item is as follows. For item 1 (“What year is it now?”), a correct answer is awarded 4 points, while an incorrect answer receives 0 points. For item 2 (“What month is it now?”), a correct answer is awarded 3 points, with 0 points for an incorrect answer. Item 4 (“About what time is it? within an hour”) is awarded 3 points for a correct answer and 0 points for an incorrect answer. Item 5 (“Count backwards 20 down to 1”) assesses completion accuracy. A score of 4 points is given for completion without errors, 2 points for completion with one error, and 0 points for completion with two or more errors. For item 6 (“State the twelve Chinese zodiac signs in reverse order”), the same criteria apply, with 4 points for completion without any error, 2 points for completion with one error, and 0 points for completion with two or more errors. Scores for item 3/7 (“Repeat the address given”) are deducted 2 points for each error on a first name, family name, number, road name, or city name, resulting in a score ranging from 0 to 10 points.

### Procedure

Participants were recruited from the Department of Rehabilitation Medicine of the First Affiliated Hospital, SunYat-sen University between August 2015 and July 2019. To determine inter-rater reliability, two therapists (raters A and B) who were familiar with the C-SOMC administered it to all the participants. Rater A was a physiotherapist with more than 10 years of clinical experience in stroke rehabilitation, while rater B had 1 year of clinical experience in stroke rehabilitation. The Day 1 assessment was conducted by rater A and rater B, while the Day 2 (on the subsequent day of Day 1) assessment was conducted by rater B only. Rater B was blinded to the results of assessments performed by rater A. On item 3/7, rater A chose either address a or b for participants to recall, and rater B chose either address c or d for participants to recall. There were 8 participants who were either unable or unwilling to participate in a third round of testing within a 2-day period, which was used to minimise the possible effect of spontaneous recovery (32). To assess intra-rater reliability, the C-SOMC was readministered by rater B to the remaining participants in a third round of testing on the second day. Based on previous studies, the remaining sample of 36 participants was sufficient to determine the reliability of the C-SOMC (17, 18). For item 3/7, the third round of testing instructed to patients was chosen during either address c or d but different from that of the second round. The C-SOMC was administered in a quiet room to minimize confounding factors.

### Statistical analysis

#### Sample size calculation

The sample size was calculated by an online calculator (33). As there was no previous study explore the reliability of C-SOMC, we assume the minimal acceptable reliability and the expected reliability as 0.75 and 0.90, with the significant level set at 0.05 and the power set as 80%. The minimal sample size was calculated to be 33 subjects. To draw a more robust conclusion, this study enrolled 44 in patients who have had a first episode of stroke.

#### Participants

Descriptive statistics were used to analyze the demographic and clinical characteristics of the participants in this study (*n* = 44).

#### Reliability

The inter-rater and intra-rater reliabilities were analysed using intraclass correlation coefficient, paired sample tests, and Bland–Altman plots.

(1) Intraclass Correlation Coefficient

To minimise the effects of spontaneous recovery and learning effect on the results, we used ICC Model 3 (Raters in the study are the specific raters of interest and fixed) to quantify the degrees of inter-rater and intra-rater reliability (34). To assess inter-rater reliability, ICC Model 3,2 (two-way mixed effects, absolute agreement, multiple raters) was used to analyze the two sets of C-SOMC data obtained though administration of the test by raters A and B at the same day. To assess intra-rater reliability, ICC Model 3,1 (two-way mixed effects, absolute agreement, single rater) was used to analyze the two sets of C-SOMC data obtained by administration of the test by rater B at two different times in 2 consecutive days. Based on a previous study, ICC values of <0.50, 0.50–0.75, 0.75–0.90, and >0.90 are considered to respectively indicate poor, moderate, good and excellent reliability (35). For the calculation of the 95% CI of ICC (35), please find it as below:

$$95\text{\%}\;\mathrm{CI}=\mathrm{ICC}\pm t(df,0.025)\times \sqrt{\mathrm{Var}(\mathrm{ICC})*}$$

where:

*t*(*df*, 0.025) is the *t*-statistic with degrees of freedom (*df*) and a two-tailed alpha of 0.025, Var(ICC) is the variance of the ICC, calculated as:

$$\mathrm{Var}(\mathrm{ICC})=\frac{(1-\mathrm{ICC})2\cdot (1+(k-1)\cdot \mathrm{ICC})2}{k^{2}\times (n-1)}$$

where:

*n* is the number of subjects; *k* is the number of raters.

In this study, the 95% CI of ICC was calculated by the SPSS directly.

The minimal detectable change (MDC) is used to represent the real change in the test rather than the measurement error (36). It was calculated by the following formula: ${\mathrm{MDC}}_{95}=1.96\times$ $\mathrm{SDpool}\times \sqrt{2\times (1-\mathrm{ICC})}$, where the SDpool is the mean SD of the SOMC scores on the two time points and the ICC is the intra-rater reliability coefficient.

(2) Paired sample *t*-test

The paired sample *t*-test will be used to assess the difference in inter-rater (rater A vs. rater B) and intra-rater (rater B's Day 1 and rater B's Day 2) assessment of C-SOMC results. The *t*-statistic is calculated using the following formula:

$$t=\frac{\bar{D}}{8_{D}/\sqrt{n^{2}}}$$

where:

8_D_ is the standard deviation of the differences.

$$8_{D\;}=\sqrt{\frac{\sum {\left(D-\bar{D}\right)}^{2}}{n-1}}$$

(3) Bland–Altman plot

To provide a more detailed analysis of the differences between scores obtained by different raters or by the same rater at different times, a Bland–Altman plot is used to compare the mean differences and 95% limits of agreement (LOA) between the total C-SOMC scores. The 95% LOA is similar to the minimal detectable change (37) and sets a threshold to distinguish real biological change (such as the spontaneous recovery of impairment) from measurement noise (38). A narrow 95% LOA signifies that the measurement tool can detect more subtle biological changes occurring over time or with an intervention (37, 39). The mean difference is calculated as the average difference between 2 assessments of the same individuals. The upper and lower boundaries of the 95%LOA are obtained by calculating the mean values from the 2 assessment sessions and adding or subtracting 1.96 standard deviations (SD) (40). A smaller mean difference and 95%LOA indicates better agreement.

All statistical analyses were generated using SPSS version 20.0 (IBM, Inc., Armonk, NY, USA). All tests were two-tailed, and a significance level of *p*-value <0.05 was used to indicate statistical significance.

## Results

### Demographics

This study enrolled 44 participants who had a first episode of stroke (ischemic, *n* = 38; haemorrhagic, *n* = 6), of whom 31 were men and 13 were women. The mean age of the participants was 59.05 ± 10.79 years, with a range of 40–80 years. Details of the demographic and clinical characteristics of the participants are provided in Table 1.

**Table 1:** Characteristics of the study participants (*n* = 44).

Variable	Inter-rater study Sample *n* = 44	Intra-rater study Sample *n* = 36
	Values	Values
Age (years)	59.05 ± 10.79 (40–80)	59.03 ± 9.92 (40–80)
Sex		
Male (%)	31 (70.5)	25 (69.4)
Female (%)	13 (29.5)	11 (30.6)
Stroke type		
Ischemic (%)	38 (86.4)	31 (86.1)
Hemorrhagic (%)	6 (13.6)	5 (13.9)
Affected side		
Right	18 (40.9)	17 (47.2)
Left	26 (59.1)	19 (52.8)
Education (years)	9.27 ± 3.961 (0–15)	9.22 ± 3.689 (5–15)
Bachelor and above (≥15)	4 (9.1)	2 (5.6)
Junior college (14)	8 (18.2)	7 (19.4)
Senior high school (11)	6 (13.6)	6 (16.7)
Technical secondary school (11)	3 (6.8)	2 (5.6)
Junior high school (8)	9 (20.5)	7 (19.4)
Primary school (5)	13 (29.5)	12 (33.3)
Illiteracy (0)	1 (2.3)	0 (0)
MMSE	25.91 ± 4.96 (6–30)	25.64 ± 5.30 (6–30)
C-SOMC	A/B	B1/B2
24–28	20 (45.5)/19 (43.2)	17 (47.2)/13 (36.1)
19–23	12 (27.3)/12 (27.3)	9 (25)/12 (33.3)
0–18	12 (27.3)/13 (29.5)	10 (27.8)/11 (30.6)

Values were mean ± SD (range) or *n* (%).

A, rater A; B, rater B; B1, the first round of evaluations by rater B; B2, the second round of evaluations by rater B.

The C-SOMC total and item performance scores obtained by the different raters are summarised in Table 2. The data from the re-evaluation of 36 participants by rater B at separate times were pooled to assess the intra-rater reliability. The demographic information for these 36 participants is also provided in Table 1. The C-SOMC total and item performance scores for these 36 participants are shown in Table 3.

**Table 2:** C-SOMC inter-rater reliability (*n* = 44).

Rater	Total		Item 1		Item 2		Item 4		Item 5		Item 6		Item 3/7	
	A	B	A	B	A	B	A	B	A	B	A	B	A	B
Mean	20.77	20.75	3.64	3.73	2.93	2.86	2.93	2.93	3.73	3.73	0.32	0.45	7.14	6.77
SD	5.039	4.998	1.163	1.020	0.452	0.632	0.452	0.452	0.924	0.924	1.052	1.210	3.296	3.026
Range	3–28	3–28	0–4	0–4	0–3	0–3	0–3	0–3	0–4	0–4	0–4	0–4	0–10	0–10
ICC	0.959^a^		0.918^a^		0.796		1.000^a^		0.942^a^		0.905^a^		0.913^a^	
95% CI	0.926–0.978		0.849–0.955		0.627–0.889		1.000–1.000		0.894–0.969		0.826–0.948		0.840–0.952	
*P*	0.000		0.000		0.000		0.000		0.000		0.000		0.000	

ICC, intraclass correlation coefficient; CI, confidence interval; A, rater A; B, rater B.

^a^ Excellent correlation.

*p* < 0.05 indicates significant correlations.

**Table 3:** C-SOMC intra-rater reliability (*n* = 36).

Rater	Total		Item 1		Item 2		Item 4		Item 5		Item 6		Item 3/7	
	B1	B2	B1	B2	B1	B2	B1	B2	B1	B2	B1	B2	B1	B2
Mean	20.86	20.53	3.67	3.67	2.83	2.83	2.92	2.92	3.72	3.78	0.33	0.39	7.06	7.00
SD	5.183	5.305	1.121	1.121	0.697	0.697	0.500	0.500	0.974	0.929	1.014	1.050	3.042	3.260
Range	3–28	3–28	0–4	0–4	0–3	0–3	0–3	0–3	0–4	0–4	0–4	0–4	0–10	0–10
ICC	0.978^a^		1.000^a^		1.000^a^		1.000^a^		0.968^a^		0.973^a^		0.929^a^	
95% CI	0.956–0.988		1.000–1.000		1.000–1.000		1.000–1.000		0.938–0.984		0.948–0.986		0.865–0.965	
*P*	0.000		0.000		0.000		0.000		0.000		0.000		0.000	

ICC, intraclass correlation coefficient; CI, confidence interval; B1, the first round of evaluations by rater B; B2, the second round of evaluations by rater B.

^a^ Excellent correlation.

*p* < 0.05 indicates significant correlations.

### Inter-rater reliability

The inter-rater reliability of the C-SOMC was evaluated using data from all 44 participants. The ICC for the total score was 0.959 [95% confident interval (CI): 0.926–0.978], indicating excellent inter-rater reliability of the C-SOMC in individuals who have had a stroke. The ICC for item 2 scores was 0.796 (95% CI: 0.627–0.889), showing moderate to good interrater reliability. The ICC for the scores for items 1, 4, 5, 6, and 3/7 ranged from 0.905 to 1.000 (95% CI: 0.826–1.000), which suggested good to excellent inter-rater reliability for these items. The details of inter-rater reliability of the C-SOMC are summarised in Table 2.

Table 4 detailed the differences between the scores for total and individual items of the C-SOMC as administered by rater A (C-SOMC A) and rater B (C-SOMC B). The differences between the scores obtained by the two raters ranged from +6 to −4 (median 0.00, 95% percentiles +2 to +6), with a mean difference of 0.02 (SD 1.982; 95% CI: −0.59 to 0.61).

**Table 4:** Difference between C-SOMC results administrated by rater A and rater B (*n* = 44).

Difference score	Total	Item 1	Item 2	Item 4	Item 5	Item 6	Item 3/7
6	1						1
5	1						
4	1						2
3			1				
2	6				1		9
0	24	43	43	44	42	42	25
−2	9				1	1	6
−4	2	1				1	1

Difference score, C-SOMC A – C-SOMC B; negative number = better score administrated by rater B.

Table 5 illustrated the paired sample *t*-test for the scores of the C-SOMC obtained by two raters demonstrating no statistically significant difference (*P* > 0.05).

**Table 5:** The paired sample *t*-test of C-SOMC administered by two raters (*n* = 44) or by rater B on separate occasions (*n* = 36).

Rater	Total		Item 1		Item 2		Item 4		Item 5		Item 6		Item 3/7	
	t	*P*	t	*P*	t	*P*	t	*P*	t	*P*	t	*P*	t	*P*
A-B (inter-rater)	0.076	0.940	−1.000	0.323	1.000	0.323	/	/	0.000	1.000	−1.354	0.183	1.346	0.185
B1-B2 (intra-rater)	1.291	0.205	/	/	/	/	/	/	−1.000	0.324	−1.000	0.324	0.206	0.838

A, rater A; B, rater B; B1, the first round of evaluations by rater B; B2, the second round of evaluations by rater B.

*P* < 0.05 indicates significant difference.

**Figure 1:**
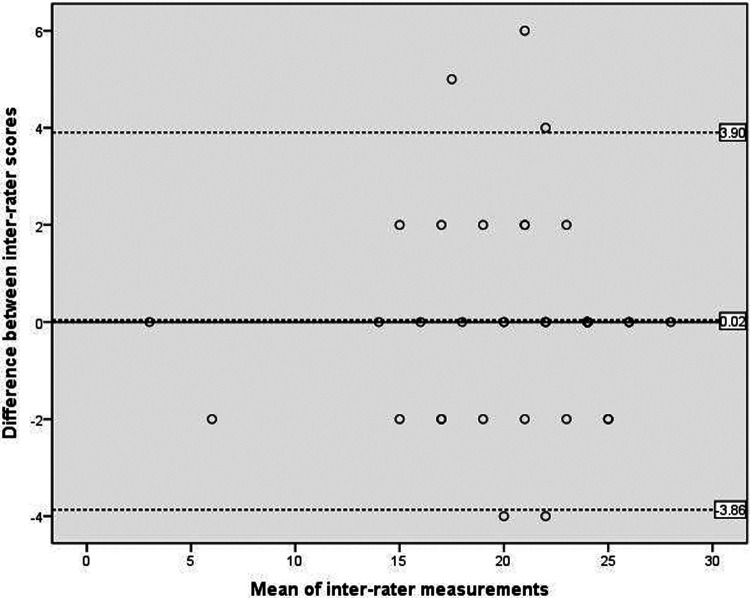
Scatterplots of the differences between two raters. The dashed bold line represented the mean difference score between two raters. The dashed lines exhibited the limits of agreement (mean ± 1.96× the standard deviation of the different score).

Figure 1 showed the analysis of inter-rater reliability. The mean difference between the scores obtained by the two raters was 0.02, which did not differ significantly from zero. The 95% LOA ranged from −3.86 to 3.90 and showed five outliers. The results showed a high level of concurrence between the C-SOMC scores obtained from different raters, corroborated by the mean difference and the 95% LOA.

### Intra-rater reliability

The intra-rater reliability of the C-SOMC was assessed using data from 36 participants who were re-evaluated by rater B at different times. The ICC for the total score was 0.978 (95% CI: 0.956–0.988), indicating excellent intra-rater reliability of the C-SOMC for individuals who have had a stroke. The ICC for item 3/7 scores was 0.929 (95% CI: 0.865–0.965), indicating good to excellent intra-rater reliability. The ICCs for the scores of items 1, 2, 4, 5, and 6 ranged from 0.968 to 1.000 (95% CI: 0.938–1.000), indicating excellent intra-rater reliability for items 1, 2, 4, 5, and 6. The minimal detectable change value with a 95% confidence threshold (MDC_95_) of SOMC score was found to be 2.14. The details of intra-rater reliability assessments of the C-SOMC are summarised in Table 3.

Table 6 showed the differences between the total and individual item scores obtained by rater B on separate occasions (C-SOMC B1 and C-SOMC B2). The differences between the scores obtained by rater B on administration of the C-SOMC at different times ranged from +4 to −4 (median 0.00, 95% percentiles +2 to +4), with a mean difference of 0.33 (SD 1.549; 95% CI: −0.17 to 0.78).

**Table 6:** Difference between C-SOMC results administrated by rater B at different occasion (*n* = 36).

Different score	Total	Item 1	Item 2	Item 4	Item 5	Item 6	Item 3/7
4	1						
2	10						10
0	20	36	36	36	35	35	19
−2	4				1	1	5
−4	1						2

Difference score, C-SOMC B1 – C-SOMC B2; negative number = better score on the second occasion.

Table 5 illustrated the paired *t*-test for the comparing C-SOMC scores obtained by rater B on separate occasions demonstrating no statistically significant difference (*P* > 0.05).

**Figure 2:**
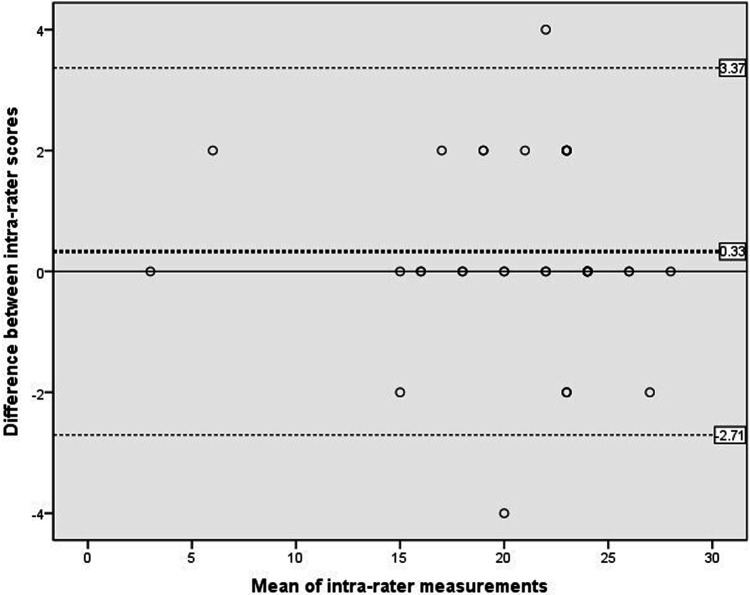
Scatterplots of the differences between two measurements by the same rater at different time points. The dashed bold line represented the mean difference score between two raters. The dashed lines exhibited the limits of agreement (mean ± 1.96× the standard deviation of the different score).

Figure 2 showed the analysis of intra-rater reliability. The mean difference between the two evaluation scores obtained by rater B was 0.33, which did not differ significantly from zero. The 95%LOA ranged from −2.71 to 3.37 and showed two outliers. The findings indicated a high level of concurrence between the C-SOMC assessments conducted by the same rater at different time points, as corroborated by the mean difference and the 95% LOA.

## Discussion

The aim of this study was to evaluate the reliability of the C-SOMC when used to assess individuals who have had a first episode of stroke. Our results indicated that the total C-SOMC scores and all individual items demonstrated good to excellent inter-rater and intra-rater reliability. There was no significant difference of C-SOMC score when assessed by different raters or by the same rater at different times. Furthermore, analyses with a Bland–Altman plot demonstrated that both the inter-rater and intra-rater evaluations showed small mean differences and 95%LOA ranges, indicating strong agreement. Therefore, the C-SOMC is a reliable test for screening cognitive impairment in individuals who have had a stroke.

The inter-rater reliability analysis conducted in our study demonstrated excellent reliability for the total score of the C-SOMC. A previous study by David et al. showed good inter-rater reliability (*k* = 0.63) of the SOMC in geriatric patients older than 75 years who presented to the emergency department (41). Age can be a significant factor contribute to the difference in the inter-rater reliability between the 2 studies. Compared with our study (mean age = 59.0 years old), older adults (e.g., >75 years) in David’ study may have lower cognitive reserve, making their cognitive performance more susceptible to fluctuations and measurement errors. Age-related changes in brain structure and function, including reduced processing speed, working memory capacity, and attention control, are well-documented in cognitive aging literature (42–44). Moreover, older adults may experience greater fatigue during testing, have more difficulty with test instructions, or show increased sensitivity to environmental distractions, all of which can contribute to measurement inconsistency across raters (45). In addition, geriatric patients in the emergency setting may have more variable cognitive states due to acute medical conditions or delirium (46). Our results also indicated excellent inter-rater reliability for items 1,4,5,6, and 3/7 and good inter-rater reliability for item 2. Although item 2 did not show excellent inter-rater reliability, a comparison of the scores obtained by rater A and rater B showed a difference in the assessment of only one participant. Additionally, the differences between the C-SOMC scores obtained for the same participant by rater A and rater B were mainly reflected in item 3/7. Twelve of the 44 subjects performed better on the item 3/7 when evaluated by rater A in the first assessment, which may be attributed to the participants being impatient or less nervous and anxious during the second round of testing on the same day. Seven of the 44 subjects performed better in item 3/7 when evaluated by rater B within one day, which could be attributed to a learning effect. Overall, our results demonstrated excellent inter-rater reliability for the total and individual item scores of the C-SOMC.

Our intra-rater reliability analysis indicated excellent reliability for both the total C-SOMC and individual item scores when the test was administered to the same participant by the same rater at different time points. Several studies have previously evaluated the intra-rater reliability of the SOMC. Davous et al. reported good intra-rater reliability of the SOMC when administered by the same rater during 18 patients with Alzheimer's disease at different evaluations done one month apart (47). Wade and Vergis investigated the intra-observer reliability of the SOMC in 38 people with a variety of neurological diseases and discovered that the SOMC was reasonably reliable when applied by one rater twice over an interval of one week (17). We administered the C-SOMC twice within a shorter time interval of one day to 36 patients with a first stroke. The higher intra-rater reliability shown in our study could be due to the fact that we included individuals with less severe cognitive impairment. Additionally, our results indicated excellent intra-rater reliability for all the individual items.

In this study, the MDC_95_ was calculated to be 2.14. The mean difference of SOMC score between the two different time points was less than 2.14, which indicated that the inter-rater and intra-rater mean difference of SOMC was mainly contributed by the measurement error rather than the real change of cognitive function. Thus, the cognitive function among the subjects was stable in this study. The variances of cognitive function due to the spontaneous recovery may not make a significant impact on our result.

The differences between the C-SOMC scores for the same participant as evaluated by rater B at different time points, were mainly from the scores on item 3/7. Ten of the 36 subjects performed better on item 3/7 during the first assessment by rater B. All participants gained two more points on the second assessment, which could be due to the participants being impatient or less nervous and anxious during the third assessment done in the span of two days. Seven of the 36 subjects showed an improvement in item 3/7 during the second assessment by rater B, which could be attributed to a learning effect. In conclusion, our results demonstrated excellent intra-rater reliability for the C-SOMC and its individual items.

Our inter-rater and intra-rater analyses showed small mean differences and acceptable 95% LOA ranges, indicating good reliability of the C-SOMC. While most measurements fell within the 95% LOA, a small percentage of participants in the inter-rater (11.4%, 5 of 44) and intra-rater (5.6%, 2 of 36) analyses fell outside these limits. The Bland-Altman plots revealed a greater 95% LOA range in the inter-rater plot compared to the intra-rater plot, suggesting slightly lower inter-rater agreement than intra-rater agreement, which was an expected pattern in reliability studies. Nevertheless, both analyses demonstrated that the C-SOMC was a stable evaluation tool. The LOA in Bland-Altman analysis is conceptually similar to minimal detectable change (39), which helps distinguish true biological differences from measurement error (48). Our finding that 88.6%–94.4% of points fell within the 95% LOA indicated that the observed differences between measurements were primarily due to expected measurement error rather than actual changes in cognitive function (38). Thus, our results suggested that the C-SOMC is sufficiently sensitive to detect meaningful changes in cognitive function among stroke patients.

In conclusion, the C-SOMC demonstrated excellent inter-rater and intra-rater reliability when administered to individuals who have had a stroke. Most participants in this study showed good orientation and attention due to superior performance on items 1, 2, 4, and 5. Some had suboptimal scores on item 3/7, suggesting that they may have memory impairment. Many participants in this study had poor performance on item 6, and most participants reported that they have never tried to remember the order of the twelve Chinese zodiac animals and could not say it in order even before their stroke. The revision of item 6 may be required in the future version of the C-SOMC.

### Clinical implication

The SOMC test is a valuable tool for assessing cognitive function, particularly in stroke populations, which comprising only 6 items (49). All items are easy to understand and administer, minimizing the risk of misunderstandings (50, 51). Its simplicity and brevity make it particularly suitable for stroke patients, who often face challenges with more complex cognitive assessments. For instance, stroke-related impairments such as hemiplegic paralysis, visual impairment, agraphia, and dyslexia can hinder the completion of common cognitive tests like the MMSE and MoCA, which require reading and writing tasks (27, 28, 52). The SOMC, however, can be completed verbally at the patient's bedside, making it accessible even in the acute phase of stroke.

Clinically, the SOMC has demonstrated significant predictive validity. Lucke et al. (53) reported that the SOMC was an independent predictor of adverse outcomes, including hospital length of stay, new institutionalization, and in-hospital mortality. Madison et al. (54) further highlighted the SOMC's importance by noting that its scores were the only acute clinical data significantly predicting communication, memory, and thinking abilities. This underscores the SOMC's potential as a prioritized screening measure for assessing acute neurologic status and cognitive dysfunction, guiding post-stroke rehabilitation recommendations.

This study has some limitations. First, this study only assessed the cognitive function level among the subjects with stroke, without evaluating other related factors such as stroke severity or emotional status. To achieve a comprehensive understanding of the factors affecting cognitive function, future cross-sectional studies should incorporate additional assessments, such as the National Institutes of Health Stroke Scale and Stroke Impact Scale, which cover a broader range of domains. Second, the study conducted three repeated measures of C-SOMC within 2 days potentially introducing a learning effect that should be considered when interpreting the results. Third, this study did not categorize the participants according to the cognitive impairment or chronicity of their stroke conditions. As a result, the findings and conclusions drawn from this study may only be relevant and applicable to individuals who possess a comparable level of cognitive function to those included in the study. Future research should consider incorporating a more diverse range of subjects with varying degrees of stroke severity and chronicity to enhance the generalizability and applicability of the results. At last, further research should explore the comprehensive psychometric characteristics of the instrument in patients with different degrees of cognitive impairment.

## Conclusion

In conclusion, our preliminary findings suggest that the C-SOMC has excellent inter-rater and intra-rater reliability when administered to people who have had a stroke. Thus, the C-SOMC is a valuable tool for measuring cognitive impairment in people who have had a stroke.

## Data Availability

The raw data supporting the conclusions of this article will be made available by the authors, without undue reservation.
