# Supplementary material for: Inter-rater and intra-rater reliability of the Chinese version of the short orientation–memory–concentration test in people with stroke
Source: Front Rehabil Sci. 2025 Aug 5;6:1614305. doi: 10.3389/fresc.2025.1614305 (PMC12361128; doi:10.3389/fresc.2025.1614305)
Supplement: Supplementary file 1 [file Datasheet1.pdf]

Appendix 1

中文版短期定向记忆注意力测试

患者姓名：　　　　　　　　　　　　　　　　　住院号：

指令		日期			
**1. 今年是哪一年？**		0 或 4 分			
		患者答案			
**2. 现在是几月份？**		0 或 3 分			
		患者答案			
**3. 重复下面的地址和称呼（选择一个）**<br>a. 李伟　b. 王军　c. 张华　d. 刘波<br>天河路 42 号　虹桥路 34 号　和平路 26 号　长安街 18 号<br>广州　上海　天津　北京<br>**试着记住，在测试结束时会再问你。**					
**4. 现在大概是几点？**<br>（*误差在一个小时内。*）		0 或 3 分			
		患者答案			
**5. 请从 20 倒数到 1。**<br>（*每个错误扣 2 分。*）<br>20 19 18 17 16 15 14 13 12 11 10 9 8 7 6 5 4 3 2 1		0　2　4 分			
**6. 请将十二生肖按照倒序说出来。**<br>（*每个错误扣 2 分。*）<br>猪 狗 鸡 猴 羊 马 蛇 龙 兔 虎 牛 鼠		0　2　4 分			
**7. 请重复刚才要你记住的人名和地址。**<br>（*每个错误扣 2 分。*）		0　2　4　6　8　10 分			
		所给地址（a, b, c, d）			
		**总分**	/28	/28	/28
