# Supplementary material for: Inter-rater and intra-rater reliability of the Chinese version of the short orientation–memory–concentration test in people with stroke
Source: Front Rehabil Sci. 2025 Aug 5;6:1614305. doi: 10.3389/fresc.2025.1614305 (PMC12361128; doi:10.3389/fresc.2025.1614305)
Supplement: Supplementary file 2 [file Datasheet2.pdf]

Appendix 2

The Chinese version of the Short Orientation Memory and Concentration Test

Patient's name： Hospital number：

Instruction		Date			
**1. What year is it now?**		Score 0 or 4			
		Patient's answer			
**2. What month is it now?**		Score 0 or 3			
		Patient's answer			
**3. Repeat this address (choose one):**<br>a. Wei Li b. Jun Wang c. Hua Zhang d. Bo Liu<br>42/Tian He Road 34/Hong Qiao Road 26/ He Ping Road 18/Chang An Street<br>Guangzhou Shanghai Tianjin Beijing<br>**Try to remember this. I'll ask you to recall it at the end of the test.**					
**4.** About what time is it?<br>（*within an hour.*）		Score 0 or 3			
		Patient's answer			
**5.** Count backwards 20 down to 1**.**<br>（*Two points off for each error.*）<br>20 19 18 17 16 15 14 13 12 11 10 9 8 7 6 5 4 3 2 1		Score 0, 2, 4			
**6.** Say the Twelve Chinese Zodiac Sign in reverse order.<br>（*Two points off for each error.*）<br>Pig Dog Chicken Monkey Sheep Horse<br>Snake Dragon Rabbit Tiger Cow Rat		Score 0, 2, 4			
**7. Repeat the address given.**<br>（*Two points off for each error.*）		Score 0, 2, 4, 6, 8, 10			
		Address given（a, b, c, d）			
		**Total Score**	/28	/28	/28
